# The role of Hrd1 in ultraviolet (UV) radiation induced photoaging

**DOI:** 10.18632/aging.103851

**Published:** 2020-11-09

**Authors:** Yi Jin, Xianye Cheng, Xin Huang, Fan Ding, Sae Rom Lee, Fengdi Wang, Xiaoyi Lu, Dongming Su, Bin Chen

**Affiliations:** 1 Department of Dermatology and Venereology, The First Affiliated Hospital of Nanjing Medical University, Nanjing 210029, Jiangsu Province, China; 2 Department of Pediatric and Preventive Dentistry, Affiliated Hospital of Stomatology, Nanjing Medical University, Nanjing 210029, China; 3 Center of Metabolic Disease Research, Nanjing Medical University, Nanjing 210029, Jiangsu Province, China

**Keywords:** Hrd1, IGF-1R, UV, photoaging, collagen

## Abstract

The purpose of the present study was to evaluate the role of Hrd1 in the ultraviolet (UV) radiation induced photoaging and explore its potential mechanism. The nude mice were exposed to the UVA/UVB irradiation for 10 weeks. The animals were subcutaneously injected with AAV5-NC, Hrd1-shRNA-AAV5, or Hrd1-overexpression-AAV5. The HSF cells were also transfected with Ad-NC, Ad-shRNA-Hrd1, or Ad-Hrd1, and irradiated by UVA/UVB stimulation. The clinical skin samples were harvested for detecting Hrd1 and IGF-1R expressions. As a result, the knockdown of Hrd1 attenuated the histopathological alteration and collagen degradation in UV-induced nude mice. The inhibition of Hrd1 by Hrd1-shRNA-AAV5 and Ad-shRNA-Hrd1 inhibited the Hrd1 expression and promoted IGF-1R, Type I collagen and type III collagen in mice and HSF cells. The overexpression of Hrd1 exerted the reverse effect. The Co-IP assay also indicated the interaction between Hrd1 and IGF-1R. Hrd1-mediated IGF-1R downregulation and collagen degradation were also observed in clinical skin samples. In conclusion, the present results demonstrated that Hrd1 degraded IGF-1R and collagen formation in UV-induced photoaging.

## INTRODUCTION

Skin aging is caused by intrinsic (regular aging process) and extrinsic aging. Extrinsic aging is owing to the chronic overexposure to ultraviolet (UV) radiation. The accumulating damage induced by UV, especially Ultraviolet B (UVB) and Ultraviolet A (UVA) radiation, leads to topical premature skin aging which is also called photoaging. Photoaging is characterized by destroyed epidermis/dermis structure, hyperpigmentation, laxity, and wrinkle formation [1]. Photoaging deeply affects our quality of life and is a heavy burden to society. Whereas the pathogenesis of photoaging remains not fully understood, thus, it is urgent for us to investigate the underlying mechanism of photoaging.

Collagen fiber, the main structure protein in human skin, accounting for 75% dry weight of dermis. As its major component, Type I and type III collagen are produced by skin fibroblasts. The degradation and absence of collagen fibers are prominently responsible for photoaging progression [2]. Insulin-like growth factor-1(IGF-1) is the growth factor and growth promoting factor. Upon binding with insulin-like growth factor-1 receiver (IGF-1R), IGF-1 exerts its biological effect and activates receptor’s tyrosine kinase activity, which consequently promotes the phosphatidylinositol-3-kinase (PI3K)/protein kinase B(Akt) pathway or mitogen activated protein kinase (MAPK) cascade [3]. Growing evidence has emerged indicating that IGF-1 augments the collagen synthesis via the IGF-1R signaling [4]. Ishikura Kinoshita et al elicited that IGF-1 directly stimulated the synthesis of Type I and type III collagen in skin fibroblasts in vitro [5]. It was proposed that IGF-1R was expressed in fibroblasts of scar tissue [6]. Type I and type III collagen were synthesized by human skin fibroblasts, which was governed by IGF-1R [7]. Thus, it was assumed that IGF-1R might be the critical modulator for collagen generation in photoaging skin.

It is widely acknowledged that Endoplasmic reticulum-associated degradation (ERAD) is responsible for recognizing and degrading misfolded proteins which cause cellular stress. The occurrence of ERAD requires ubiquitin proteasome cascade [8]. The ubiquitin ligases modify substrates by attaching various protein ubiquitin, thus the substrates are tagged and degraded by cytosolic 26S proteasome in cytoplasm [9]. Hrd1, namely hydroxymethylglutaryl coenzyme A reductase degrades protein 1, belongs to E3 ligase family. Hrd1 is generally regarded as a ring finger domain E3 family located in endoplasmic reticulum [10]. Hrd1 is capable of decomposing the misfolded or unfolded endoplasmic reticulum accumulation to protect cells against endoplasmic reticulum stress [11]. It was elicited that Hrd1 degraded IGF-1R and induced apoptosis in cardiomyocyte [12]. Hrd1 also suppressed the growth and metastasis of breast cancer cells by accelerating IGF-1R degradation [13]. Thus, it was assumed that IGF-1R might be the substrate of Hrd1 and Hrd1 might exert its biological effect via the degradation of IGF-1R. Our previous investigation displayed that Hrd1 was upregulated in sun-exposed skin, especially in elder donors. Whereas the mechanism of Hrd1 in UV-induced photoaging remains not fully elucidated. The present study was conducted to evaluate the effect of Hrd1 on UV-induced photoaging and explore its mechanism.

## RESULTS

### The effect of Hrd1 transfection on the histopathological alteration in UV-induced nude mice

**Figure 1:**
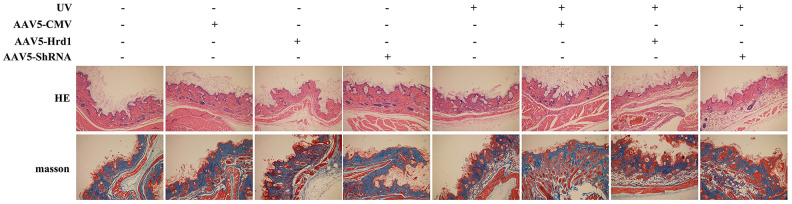
**The effect of Hrd1 transfection on the histopathological alteration in UV-induced nude mice by HE staining and Masson’s trichrome staining.**

As shown in Figure 1, the intact structure of the epidermis was observed in in AAV5-NC treated mice. Obvious epidermal papilla, dermal papilla and compact wavy fibrous tissue were visualized. On the contrary, UV irradiation caused incomplete structure of epidermis/dermis, loosed fiber distribution, decreased collagen content, irregular sebaceous gland and infiltrated inflammatory cell compared with those in AAV5-NC group. The administration of Hrd1-shRNA-AAV5 contributed to the attenuation of pathological damage, while the administration of Hrd1-overexpression-AAV5 deteriorated the pathological alteration. Moreover, the treatment with Hrd1-overexpression-AAV5 also led to the pathological lesion compared with control mice.

Additionally, the UV stimulation conduced to the reduction of collagen fiber network, loosed fiber distribution, disarranged and dysplastic collagen fiber in Masson’s trichrome staining compared with AAV5-NC mice. Besides, the treatment with Hrd1-shRNA-AAV5 and Hrd1-overexpression-AAV5 augmented and inhibited the collagen degradation, respectively. The Hrd1-overexpression-AAV5 administration also resulted in the reduced bundles of collagen fibers.

### The effect of Hrd1 transfection on protein expressions of Hrd1, IGF-1R by immunohistochemistry in mice

**Figure 2:**
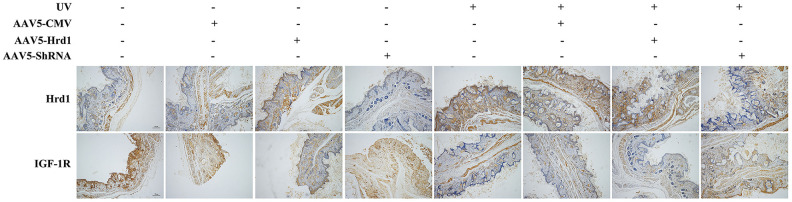
**The effect of Hrd1 transfection on protein expressions of Hrd1, IGF-1R in mice by immunohistochemistry.**

To further verify the effect of Hrd1 transfection, we performed Immunohistochemistry staining. As observed in Figure 2, UV challenge upregulated the Hrd1 expression and downregulated IGF-1R expression. The transfection with Hrd1-shRNA-AAV5 efficiently restrained Hrd1 expression and enhanced IGF-1R expression compared with UV group. Notably, the transfection with Hrd1-overexpression-AAV5 promoted Hrd1 generation and blocked the production of IGF-1R. Compared with AAV5-NC group, Hrd1-overexpression-AAV5 treatment increased the protein concentration of Hrd1 and Hrd1-shRNA-AAV5 enlarged the protein concentration of IGF-1R.

### The effect of Hrd1 transfection on protein expressions of Hrd1, IGF-1R, Type I collagen and Type III collagen by western blot in mice

**Figure 3:**
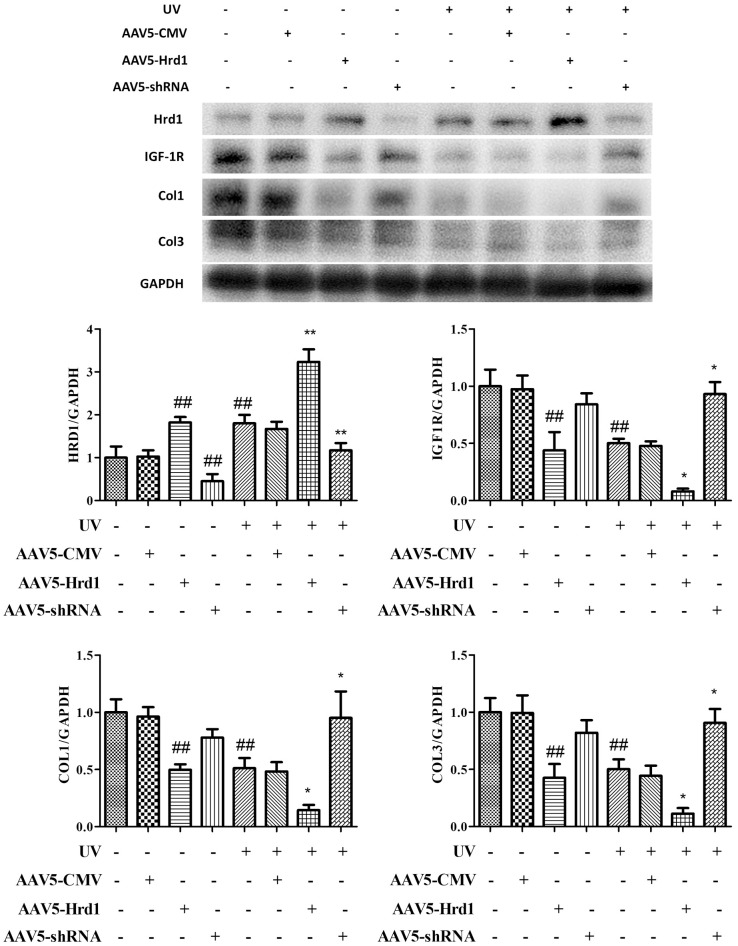
**The effect of Hrd1 transfection on protein expressions of Hrd1, IGF-1R, Type I collagen and Type III collagen by western blot in mice.** The results were presented as mean ± SD. ^##^< 0.01 compared with control group. *P < 0.05, **P < 0.01 compared with UV group.

As observed in Figure 3, the administration of Hrd1-shRNA-AAV5 downregulated the expression of Hrd1, upregulated the expressions of IGF-1R, Type I Collagen and Type III Collagen. Nonetheless, the administration of Hrd1-overexpression-AAV5 upregulated the expression of Hrd1 and downregulated the expressions of IGF-1R, Type I Collagen and Type III Collagen. UV irradiation upregulated Hrd1 protein level and downregulated the protein levels of IGF-1R, Type I Collagen and Type III Collagen. With the stimulation of UV irradiation, the treatment with Hrd1-shRNA-AAV5 suppressed Hrd1 activity and accelerated the generations of IGF-1R, Type I Collagen and Type III Collagen. However, the treatment with Hrd1-overexpression-AAV5 exhibited reversed effect compared with that of Hrd1-shRNA-AAV5.

### The effect of Hrd1 transfection on mRNA expressions of Hrd1, IGF-1R, Type I collagen and Type III collagen in mice

**Figure 4:**
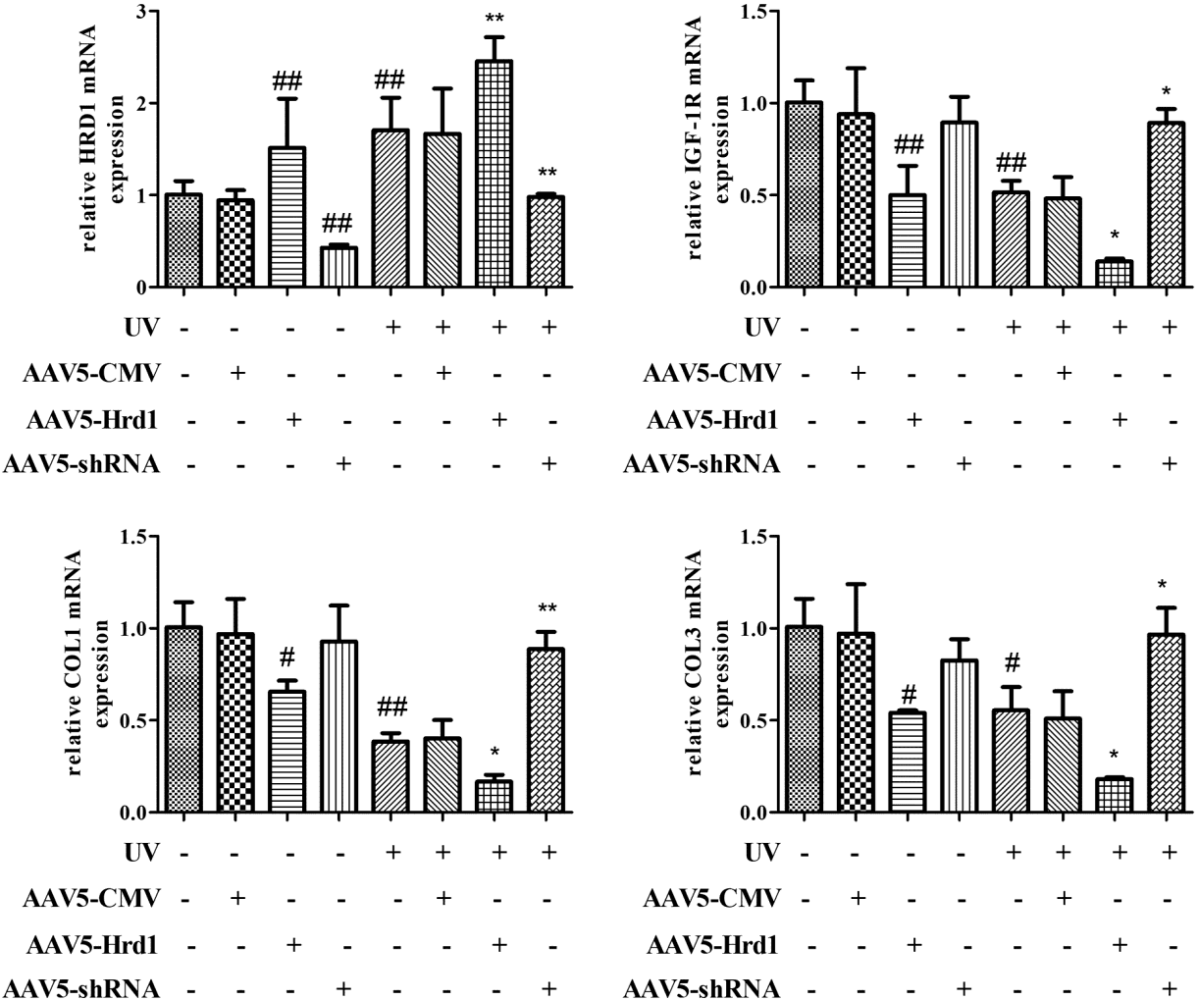
**The effect of Hrd1 transfection on mRNA expressions of Hrd1, IGF-1R, Type I collagen and Type III collagen in mice.** The results were presented as mean ± SD. ^##^< 0.01 compared with control group. *P < 0.05, **P < 0.01 compared with UV group.

qPCR assay was used to quantify the mRNA expressions of Hrd1, IGF-1R, Type I Collagen and Type III Collagen. As illustrated in Figure 4, the transfection with Hrd1-shRNA-AAV5 inhibited the transcriptional production of Hrd1, increased the transcriptional levels of IGF-1R, Type I Collagen and Type III Collagen. On the contrary, the transfection with Hrd1-overexpression-AAV5 evidently upregulated the mRNA expression of Hrd1, and downregulated the mRNA expressions of IGF-1R, Type I Collagen and Type III Collagen. UV irradiated stimulation caused the promotion of Hrd1 mRNA expression and the blockade of mRNA generations of IGF-1R, Type I Collagen and Type III Collagen. The treatment with Hrd1-shRNA-AAV5 relieved the changes induced by UV challenge. While the treatment with Hrd1-overexpression-AAV5 deteriorated the UV-caused changes.

### The effect of Hrd1 transfection on UV-Induced collagens degradation in HSF cells

**Figure 5:**
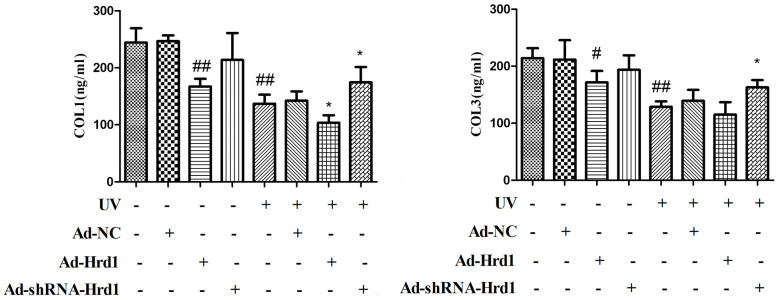
**The effect of Hrd1 transfection on UV-Induced collagens degradation in HSF cells by ELISA method.** The results were presented as mean ± SD. ^##^< 0.01 compared with control group. *P < 0.05, **P < 0.01 compared with UV group.

To estimate the effects of Hrd1 on Collagen degradation in UV-exposed HSF cells. The levels of Type I Collagen and Type III Collagen were determined using ELISA method. As depicted in Figure 5, the UV challenge contributed to the downregulation of Type I Collagen and Type III Collagen compared with that in control group. The treatment with Ad-ShRNA-Hrd1 elevated the levels of Type I Collagen(p <0.01) and Type III Collagen(p <0.05), whereas Ad-Hrd1 reduced the levels of Type I Collagen(p <0.01) and Type III Collagen(p <0.05). Compared with UV group, the HSF cells treated with Ad-ShRNA-Hrd1 notably increased the concentration of Type I Collagen(p <0.05) and Type III Collagen(p <0.05). The administration of Ad-Hrd1 evidently blocked the formation of Type I Collagen and Type III Collagen (p <0.05).

### The effect of Hrd1 transfection on the protein expressions of Hrd1, IGF-1R, Type I collagen and Type III collagen by western blot in HSF cells

**Figure 6:**
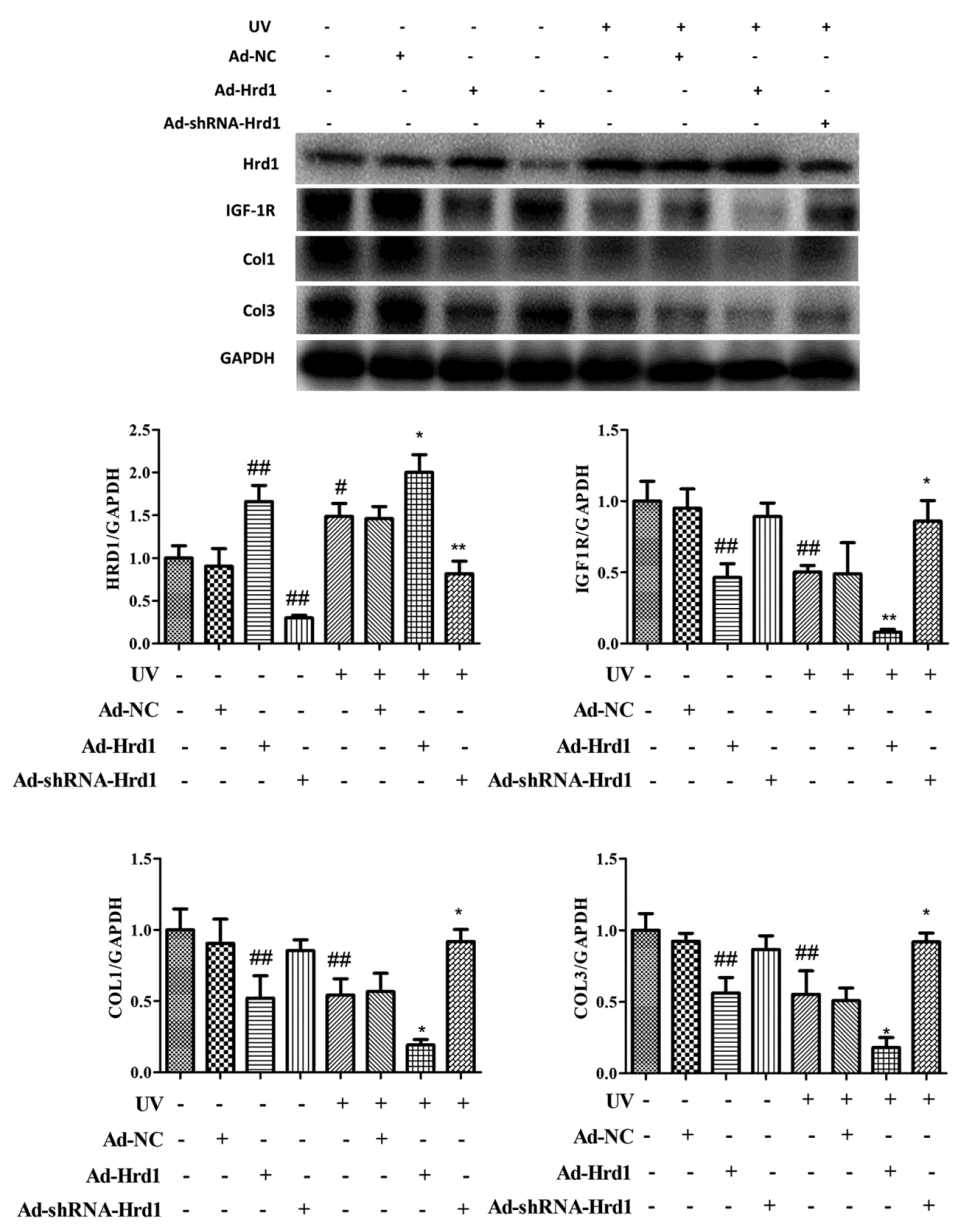
**The effect of Hrd1 transfection on the protein expressions of Hrd1, IGF-1R, Type I collagen and Type III collagen by western blot in HSF cells.** The results were presented as mean ± SD. ^#^< 0.05, ^##^< 0.01 compared with control group. *P < 0.05, **P < 0.01 compared with UV group.

Based on the above data, we further explored the molecular mechanism of Hrd1 on UV-induced HSF cells. As illustrated in Figure 6, the incubation with Ad-ShRNA-Hrd1 significantly downregulated Hrd1 expression (p <0.01) and the incubation with Ad-Hrd1 upregulated Hrd1 expression (p <0.01), which also confirmed the transfection of adenovirus on the HSF cells. Exposure to UV dramatically upregulated the expression of Hrd1 compared with the control group and Ad-NC group. To the cells exposed to UV stimulation, Of note, the treatment with Ad-ShRNA-Hrd1 also effectively downregulated Hrd1 expression (p <0.01) and the treatment with Ad-Hrd1 upregulated Hrd1 expression (p <0.05).

Additionally, as presented in Figure 6, the transfection with Ad-ShRNA-Hrd1 markedly reduced the protein level of Hrd1 and increased the protein levels of IGF-1R, Type I Collagen and Type III Collagen. Nevertheless, the transfection with Ad-Hrd1 decreased the protein levels of IGF-1R, Type I Collagen and Type III Collagen compared with those in control and Ad-NC groups. The challenged with UV irradiation effectively blocked the expressions of IGF-1R, Type I Collagen and Type III Collagen. On the contrary, under the irradiation of UV, the treatment with Ad-ShRNA-Hrd1 prominently augmented the expressions of IGF-1R, Type I Collagen and Type III Collagen (p <0.05) and treatment with Ad-Hrd1 suppressed the expressions of IGF-1R, Type I Collagen and Type III Collagen(p <0.05)

### The effect of Hrd1 transfection on the protein expressions of Hrd1 and IGF-1R by immunohistochemistry in HSF cells

**Figure 7:**
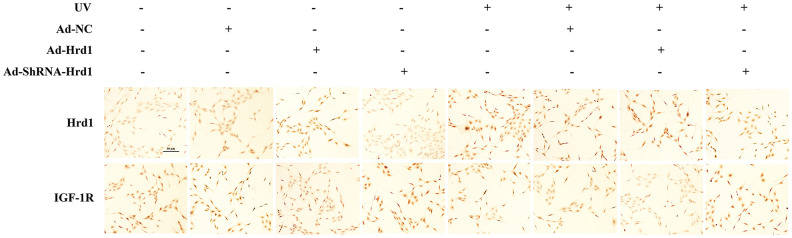
**The effect of Hrd1 transfection on the protein expressions of Hrd1 and IGF-1R by immunohistochemistry in HSF cells.**

To further evaluate the expressions of Hrd1 transfection on the expressions of Hrd1 and IGF-1R, we carried out IHC staining in UV-induced HSF cells. As revealed in Figure 7, the transfection with Ad-ShRNA-Hrd1 suppressed the expression of Hrd1 and enhanced the expressions of IGF-1R, while transfection of Ad-Hrd1 promoted Hrd1 expression and inhibited IGF-1R expression. Besides, the UV irradiation upregulated Hrd1 and downregulated IGF-1R expressions. The transfection with Ad-Hrd1 accelerated this alteration and Ad-ShRNA-Hrd1 blocked this changes. The results were in accordance with the western blot results.

### The effect of Hrd1 transfection on the mRNA expressions of Hrd1, IGF-1R, Type I collagen and Type III collagen in HSF cells

**Figure 8:**
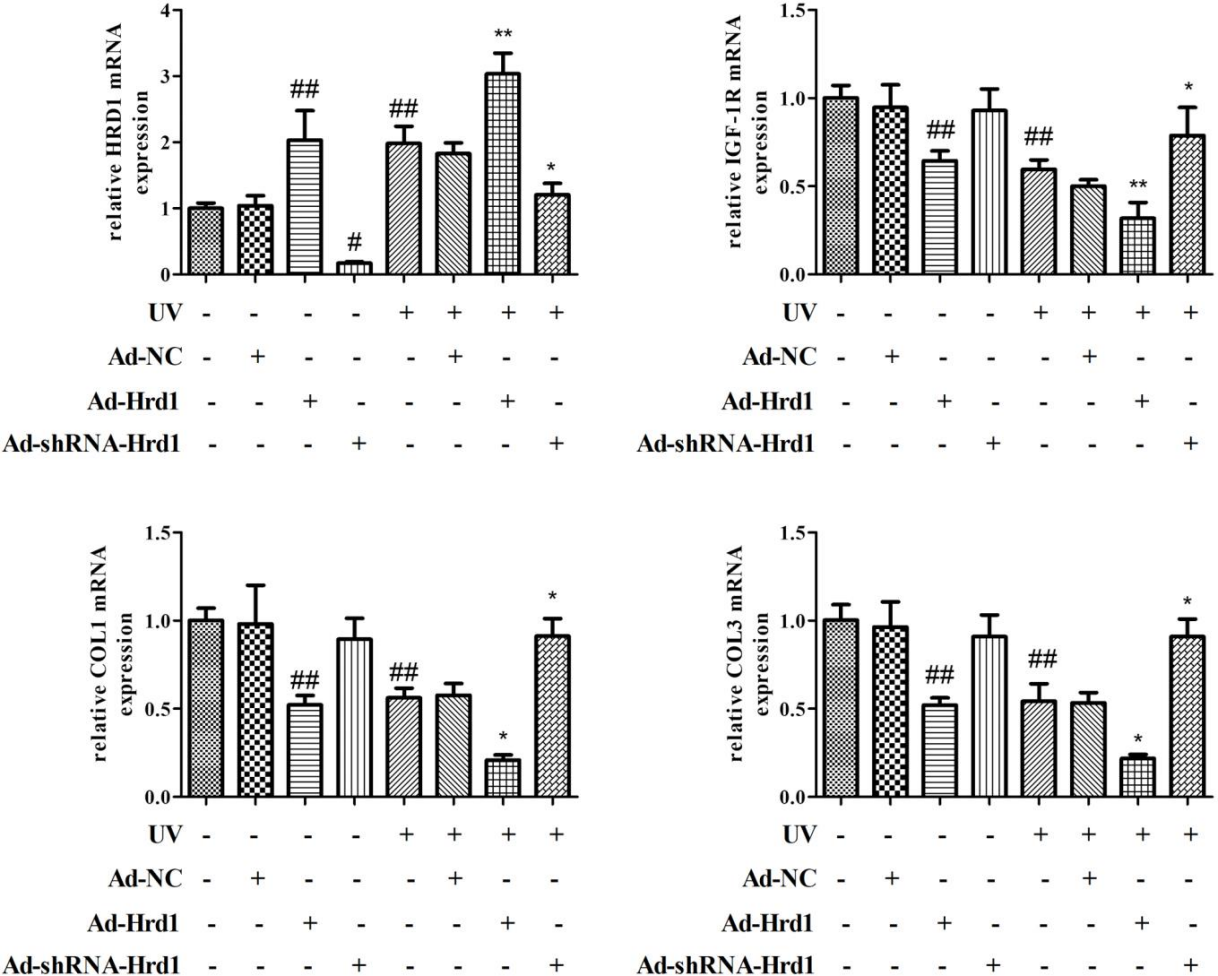
**The effect of Hrd1 transfection on the mRNA expressions of Hrd1, IGF-1R, Type I collagen and Type III collagen in HSF cells.** The results were presented as mean ± SD. ^##^< 0.01 compared with control group. *P < 0.05, **P < 0.01 compared with UV group.

Next, we detected the mRNA expressions to estimate the transcriptional effect of Hrd1 transfection. As illustrated in Figure 8, the administrations of Ad-ShRNA-Hrd1 notably inhibited Hrd1 expression and promoted IGF-1R, Type I Collagen and Type III Collagen mRNA expressions. By contrast, the administrations of Ad-Hrd1 markedly upregulated Hrd1 mRNA expression and suppressed IGF-1R, Type I Collagen and Type III Collagen mRNA expressions. UV induction increased the transcriptional level of Hrd1 and reduced the transcriptional levels of IGF-1R, Type I Collagen and Type III Collagen. The treatment of Ad-ShRNA-Hrd1 decreased the Hrd1 mRNA content and restored the mRNA contents of IGF-1R, Type I Collagen and Type III Collagen compared with those in UV group. As compared with UV-stimulated group, the treatment with Ad-Hrd1 increased Hrd1 mRNA level and reduced IGF-1R, Type I Collagen, Type III Collagen mRNA levels.

### The effect of Hrd1 transfection on Co-IP

**Figure 9:**
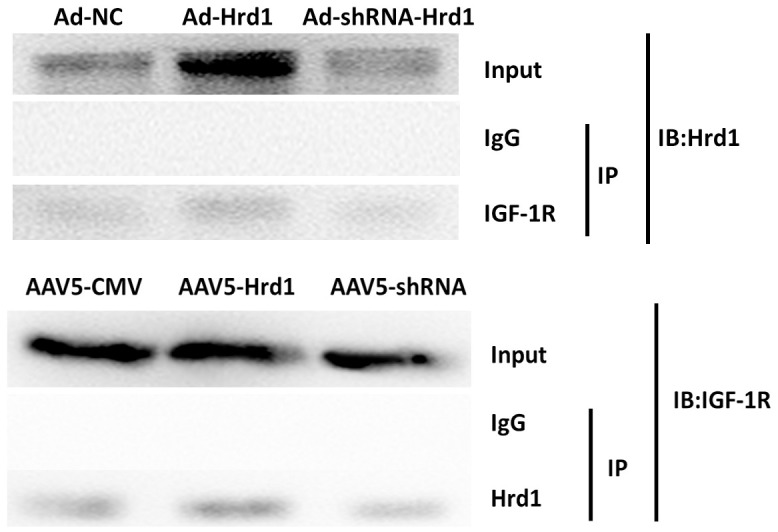
**The effect of Hrd1 transfection on Co-IP.**

Co-IP assay was applied to further investigate the cross-talk occurs between Hrd1 and IGF-1R receptor. The result showed that Hrd1 could be relatively pulled down with the treatment of anti-IGF-1R antibody. The overexpression of Hrd1 with Ad-Hrd1 improved the combination of Hrd1 and IGF-1R. Whereas the inhibition of Hrd1 with Ad-ShRNA-Hrd1 suppressed the interaction between Hrd1 and IGF-1R. Besides, the IGF-1R expression was upregulated by Ad-Hrd1 and downregulated by Ad-ShRNA-Hrd1. The co-IP detection displayed the association of Hrd1 and IGF-1R (Figure 9).

### The expressions of Hrd1, IGF-1R, Type I collagen and Type III collagen in clinical samples

**Figure 10:**
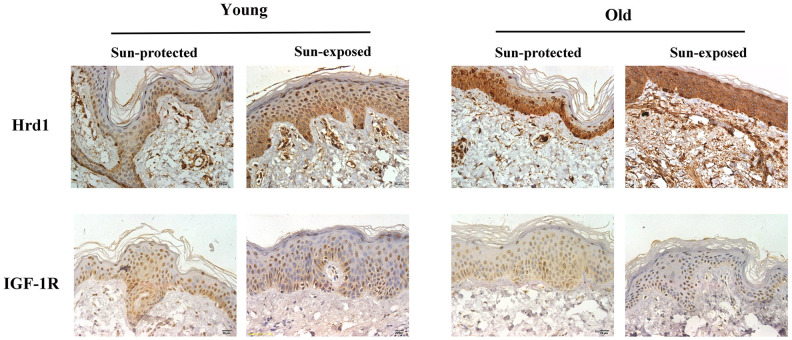
**The expressions of Hrd1 and IGF-1R by immunohistochemistry in clinical patients.**

In order to verify the Hrd1/IGF-1R expressions in clinical skin samples, the immunohistochemistry visualization was performed. As revealed in Figure 10, the sun-exposed group presented higher Hrd1 expression than sun-protected group. Nonetheless, the more enhanced IGF-1R expression in sun-protected group was observed than sun-exposed protected group.

**Figure 11:**
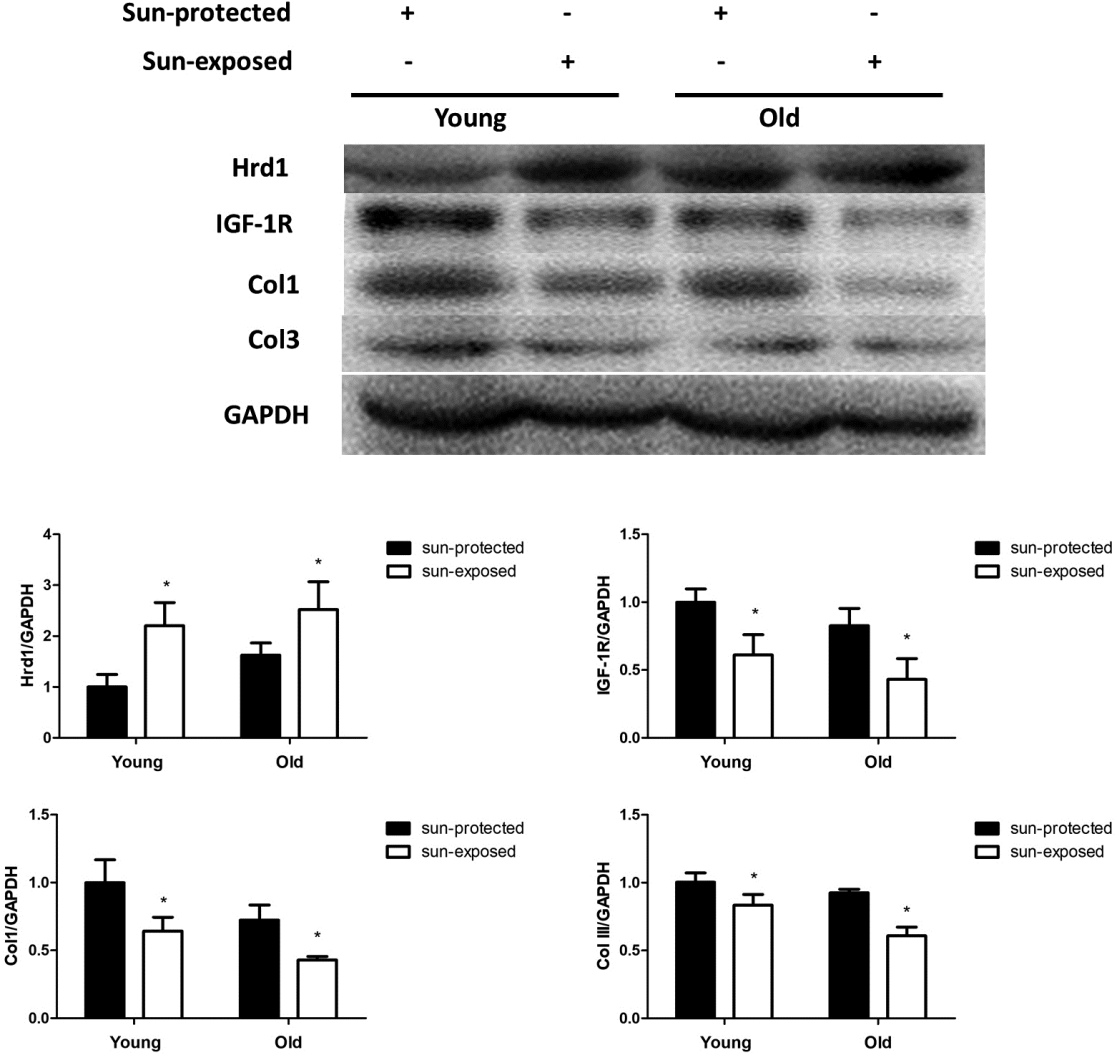
The expressions of Hrd1, IGF-1R, Type I collagen and Type III collagen by western blot in clinical samples. *P < 0.05 compared with sun-protected group.

In addition, the protein expressions of Hrd1, IGF-1R, Type I Collagen and Type III Collagen were detected by western blot and PCR. As illustrated in Figure 11, the slight more Hrd1 expression was found in old donors compared with that in young donors. The sun-exposed group exerted higher Hrd1 levels than sun-protected group both in young and old donors(p<0.05). The results indicated that Hrd1 was closely associated with photoaging. The expressions of IGF-1R, Type I Collagen and Type III Collagen in sun-exposed group were less than sun-protected group both in young and old donors (p <0.05).

**Figure 12:**
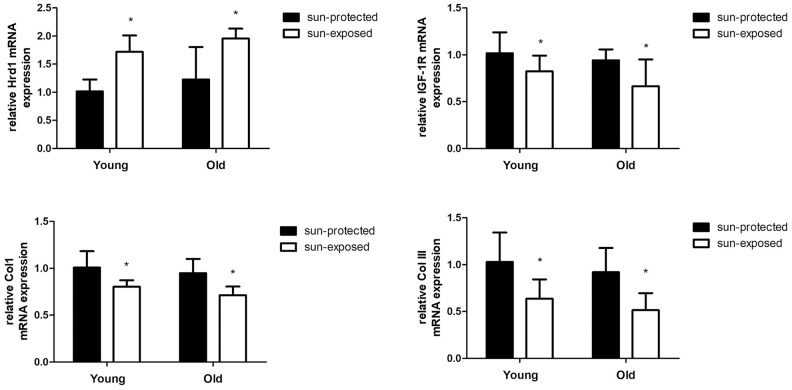
**The mRNA expressions of Hrd1, IGF-1R, Type I collagen and Type III collagen in clinical samples.** *P < 0.05 compared with sun-protected group.

Similarly, the Hrd1 mRNA in Old donors was slightly more than young donors. The sun-exposed group showed elevated Hrd1 level than sun-protected group(p <0.05). The sun-exposed group showed decreased IGF-1R, Type I Collagen and Type III Collagen expressions than sun-protected group. Our clinical data proved that Hrd1-mediated IGF-1R downregulation and collagen degradation were also observed in clinical skin samples (Figure 12).

## DISCUSSION

As the largest organ, skin protect body against a variety of physical, chemical and biological stimuli. Among the harmful factors, ultraviolet radiations (UVRs) contribute to multiple skin dysfunctions including inflammation, aging and tumor. UVRs are identified as three types: UVA (315-400 nm), UVB (280-315 nm), and UVC (100-280 nm). It is widely acknowledged that only UVA and UVB reach the surface of earth. It is noteworthy that UVB penetrates the upper layer of the dermis and causes larger damage to skin tissues than UVA [14]. In the present study, UV exposure resulted in the obvious histopathological alterations, which confirmed the successful establishment of UV-induced photoaging murine model. In our research, we showed that the knockdown of Hrd1 by AAV5 effectively ameliorated the pathological changes, which proved that Hrd1 might be a therapeutic target for UV-induced photoaging. Besides, the transfection of Ad-Hrd1 in HSF confirmed the critical effect of Hrd1 in vitro. The clinical IHC staining also confirmed that Hrd1 expressions were upregulated both in old and sun-exposed skin area. The above evidence indicated Hrd1 might be a novel target for UV-induced photoaging.

The main clinical feature of photoaging includes wrinkles, decreased elasticity, altered extracellular matrix composition, reduced collagen composition, the lack of collagen fiber and elastic fiber tissue structure. Collagen is regarded as the natural protein and functions as the major constituents of the connective tissue. Collagen consist of type I Collagen, type II Collagen, type III Collagen, and type IV Collagen. Type I Collagen is identified as the major gradient of collagen in dermis and serves as the thick fiber which maintains the structure of dermis by stiffness. Besides, Type III Collagen is the thin fiber which provides the resiliency for skin. The disarranged Collagen fibers participate in the progression of cutaneous aging induced by UV radiation [15, 16]. Thus, we assumed that UV irradiation contributed to collagen degradation in murine, cellular and clinic. Our IHC results suggested that UV irradiation contributed to the degradation of Collagen in murine model and clinical donor. The treatment with Hrd1-shRNA-AAV5 and Ad-shRNA-Hrd1 notably promoted the concentration of Type I Collagen and Type III Collagen in vivo and in vitro. The analytical data indicated that the inhibition of Hrd1 could suppress the Collagen degradation in UV-induced nude mice and HSF cells. The clinical data also confirmed that the collagen mRNA and protein expressions were also downreglated in sun-exposed area in both young and old individuals, especially in elder persons.

Matà et al elicited that IGF-I/IGF-1R upregulated collagen receptor in breast cancer [17]. The inhibition of IGF-1R was associated with the downregulation of type I collagen in dental pulps [18]. Thus, it was hypothesized that IGF-1R might be a positive modulator of collagen generation. Besides, growing evidence confirmed the crucial function of IGF-1R in skin tissues. The recovery of type I collagen and type III collagen was found to be implicated with the elevated protein level of IGF-1R in the skin of streptozotocin-induced diabetic rats [19]. The collagen formation was mediated by the IGF-1R pathway in human skin fibroblast HS68 cells under H_2_O_2_ stimulation [20]. The elevated IGF-1R level was visualized in hypertrophic scar formation [21]. Since IGF-1R was expressed in skin tissues and functioned as the mediator of collagen production, we assumed that whether IGF-1R-regulated collagen degradation could be the potential target of photoaging. Previous literature found the downregulation of IGF-1 in the UVB-induced senescence-like characteristics in human dermal fibroblasts [22]. UVA irradiation caused the downregulation of insulin-like growth factor (IGF) mRNA in photo-aged human skin cell [23]. Bentov et al proved that IGF-1R expression and activation were both inhibited in elder human skin fibroblasts rather than in younger ones. The changed IGF-1R levels were closely associated with the generation of the proliferative ability in human skin fibroblasts [24]. Therefore, IGF-1R might also be an essential modulator in photoaging skin tissues. To verify this hypothesis, we detected the IGF-1R expressions using Western blot and PCR. Our results displayed that IGF-1R was positive associated with type I collagen and type III collagen both in UV-induced nude mice and HSF cells. The suppressed IGF-1R mRNA and protein expressions were also observed in sun-exposed skin sample. Our data suggested that IGF-1R governed collagen degradation and was closely associated with the UV-caused photoaging.

Various stimuli including inflammation, oxidative stress, ischemia and irradiation contribute to the protein misfolding, which consequently cause endoplasmic reticulum stress (ERS). UVA stimulated photoaging triggers ER stress by increasing the protein levels of ER stress-associated proteins GRP78 and CHOP, as well as promoting collagen metabolism in human dermal fibroblasts [25]. Sheikh et al observed ER stress in UVB-irradiated human primary dermal fibroblasts [26]. It was verified that UVB irradiation also caused the upregulations of ER stress protein markers including CHOP, ATF4 and p-eIf2α in HS68 cells. Excessive ER stress contributes to unfolded protein response (UPR) [27]. Thus, UPR caused by UV irradiation damages the cellular homostasis, which can be relieved by Ubiquitination-related degradation of harmful protein [28]. Ubiquitin-proteasomal degradation system transfers accumulative misfolding protein to 26S proteasome, which is also known as the ER-associated protein degradation(ERAD). UV irradiation conduces to ERS in UVB/UVA exposed epidermis [29]. UVA irradiation results in ERS in UVA-induced mouse epidermal cells [30]. The ERAD activation caused by irradiation-stimulated keratinocyte exerts protective property against ERS [31].

Ubiquitination process requires ubiquitin activating enzyme(E1), ubiquitin conjugating enzyme(E2), ubiquitin ligase(E3). It is widely acknowledged that E3 ubiquitin ligase governs the specific recognition and binding between ubiquitin and protein substrate, which governs the ERAD progression [32]. Hrd1, which also known as synoviolin, is the member of E3 ubiquitin ligase family which coordinates the progression of protein degradation in ERS. Hrd1 is the Ring finger domain family E3 ligases located in endoplasmic reticulum membrane. Hrd1 constitutes the critical structural of transmembrane pore which participates in the physiological process of cytoplasmic proteasomes [33]. The E3 ubiquitin ligase Hrd1 is regarded as the component accelerating the degradation of misfolded proteins during ERAD [34]. Hrd1 exhibits multifunction in various disorders. Hrd1 augments the degradation of overexpressed p-tau to relieve Alzheimer’s disease [35]. In the synovial tissue, Hrd1 promotes the degradation of IRE1 to induce the proliferation of synovial cells which is considered as the key pathogenesis of Rheumatoid Arthritis [36]. It was also proved that Hrd1 mediated IGF-1R ubiquitination and degradation, which protect the kidney tissues in db/db mice [37]. To explore the potential mediator of IGF-1R and verify whether Hrd1 could induce the degradation of IGF-1R, we detected the expressions of Hrd1 in protein and transcriptional levels. Our experiment employed the AAV5 and adenovirus to transfection nude mice and HSF cells, respectively. We found that Hrd1 were upregulated in UV-induced skin tissues of mice and HSF cells. The inhibition of Hrd1 conduced to the upregulation of IGF-1R and increased collagen content, whereas the overexpression of Hrd1 resulted in the downregulation of IGF-1R and collagen degradation. The data confirmed the critical role of Hrd1 in UVA/UVB-induced mice and HSF cells. Hrd1 downregulated IGF-1R expression and degraded the collagen. The clinical results also proved that Hrd1 was the also upregulated in sun-exposed area, especially in elder donors. The co-IP results demonstrated the directed interaction between Hrd1 and IGF-1R.

In conclusion, our experiment displayed that Hrd1 suppressed IGF-1R and further degraded collagen in UV irradiation-induced nude mice and HSF cells, and sun-exposed skin samples. Therefore, Hrd1 might be the potential treatment target of photoaging.

## MATERIALS AND METHODS

### Reagents

AAV5-CMV-Luciferase, AAV5-u6-shRNA-P2A-Luciferase(2in1 shRNA, Primer: siRNA1:5’-TCATCAAGGTTCTGCTGTA-3’: siRNA2:5’-CCATGAGGCAGTTCAAGAA-3’,), and AAV5-CMV-Hrd1-P2A-Luciferase(Gene sequence number: NM_172230) were produced by Vigene(Jinan, China). Adenovirus: Ad-NC(empty vector), Ad-shRNA-Hrd1(The shRNA sequence targeting Hrd1: 5’-TCATCAAGGTTCTGCTGTA-3’) and Ad-Hrd1(GenBank No. NM_172230) were obtained from Vigene(Jinan, China). Dulbecco’s modified eagle medium (DMEM) was supplied from Life Technologies (Carlsbad, USA). Anti-IGF-1R antibody was obtained from abcam (ab182408, Cambridge, UK) and Santa cruz(sc-81464, CA, USA). Anti-Hrd1 antibody was obtained from Santa cruz (sc-293484, CA, USA) and abcam(ab170901, Cambridge, UK). Anti-type I collagen antibody was provided by abcam(ab34710, Cambridge, UK), anti-type III collagen antibody was supplied from Proteintech(22734-1-AP, Chicago, USA). Fetal bovine serum (FBS) was purchased from Sigma-Aldrich (St. Louis, MO, USA).

### Animals and treatments

Male and female normal nude mice at 8 weeks old were provided by Qinglongshan Animal Culture Farm(Nanjing, China). The animals were housed in specific pathogen free environment at 25 ± 2 °C with 12 h light/dark cycle. The mice had free access to the standard food and water. The experiment was approved by the Institutional Animal Care and Use Committee of Nanjing Medical University. All the efforts were made to minimize the suffering and number of animals.

The nude mice were randomly allocated into eight groups: control group, AAV5-CMV-Luciferase (AAV5-NC, or AAV5-CMV) group, AAV5-CMV-Hrd1-P2A-Luciferase(Hrd1-overexpression-AAV5, or AAV5-Hrd1) group, UV group, AAV5-NC + UV group, AAV5-CMV-Hrd1-P2A-Luciferase + UV group. AAV5-u6-shRNA-P2A-Luciferase (Hrd1-shRNA-AAV5, or AAV5-shRNA) group, AAV5-u6-shRNA-P2A-Luciferase + UV group. After the habitation, the animals were subcutaneously injected with AAV5-NC, Hrd1-shRNA-AAV5, or Hrd1-overexpression-AAV5 at the dosage of 5 × 10^11^ VG/ mice. The AAV transfection was carried out at five points with 1 cm apart at the depth of 1-2 mm on the center of the back skin. Following the injection, the needle tip was kept in the skin for a few seconds in order to avoid leakage. The needle was removed and the wound was covered by sterile cotton in order to restrain the leakage of AAV5 solution [38]. Thereafter, the UVA/UVB-induced photoaging procedure was carried out three times a week for 10 weeks. During exposure, the mice could move freely in closed box at 25 cm of the distance from the lamp to the bottom of the box. The irradiation intensity was set at UVA-15J/d(Philips, G-T60W, 365-420 nm, Amsterdam, Netherlands) and UVB-40mJ/d(Philips, PL-S9W, 290-315 nm, Amsterdam, Netherlands). The Irradiation output was monitored with a Waldmann UV-meter (Waldmann, Villigen-Schwenningen, Germany) [39].

After the irradiation, the nude mice were sacrificed. A part of skin tissues suffered for the UV stimulation at the buttock and back area were stored at -80 °C for western blot and qPCR analyses. The other skin tissues were fixed with 4% paraformaldehyde for haematoxylin and eosin (HE) staining, Masson’s trichrome staining, and immunohistochemistry (IHC).

### Histological analysis

To evaluate the histopathological alteration in photoaging mice, the H&E staining was carried out. The skin tissues were fixed by 4% paraformaldehyde and embedded in paraffin. After 4 μm section was mounted on the slides, the slides were deparaffinized, rehydrated and stained with hematoxylin and eosin solution according to the instruction.

To evaluate the collagen in skins, Masson’s trichrome staining was performed. The skin sections were deparaffinized, stained in biebrich scarlet and acid fuchsin. The slides were subsequently added with phosphomolybdic-phosphotungstic for 10 min and then aniline blue for another 5 min. After incubating with acetic acid, the sample was dehydrated.

The skin sample was observed at 400× magnification using an Olympus AX70 light microscope (Tokyo, Japan).

### Cell culture and treatments

Human skin fibroblasts were obtained from the cell bank of the Chinese Academy of Sciences, (Shanghai, China). The cells were cultured in DMEM medium supplemented with 10% FBS (Hyclone, South America), 100 IU/ml streptomycin and 100 IU/ml penicillin at the humid environment at 37°C with 5% CO_2_.

The HSF cells were assigned to control group, Ad-NC group, Ad-Hrd1 group, Ad-Hrd1 + UV group, UV group, Ad-NC + UV group, Ad-ShRNA-Hrd1 group, Ad-ShRNA-Hrd1 + UV group. 4 ×10^5^ cells were seeded on the six-well plate. 24 h later, HSF were incubated for 24 h with 2 × 10^6^ PFU Ad-NC, Ad-Hrd1, Ad-ShRNA-Hrd1 with serum-starved culture medium. The HSF cells were exposed to UVA 4J/cm^2^ and UVB 20mJ/cm^2^ irradiation. The irradiation distance between the UV source and HSF cell was 15 cm and the radiometer was also employed to control the irradiation concentration. After another incubation for 24 h with normal medium, the cells were harvested for western blot, qPCR and co-IP test.

### Collection of clinical skin samples

The clinical procedure was carried out in accordance with the Declaration of Helsinki principle. Samples of human skin(n=20) were obtained from surgeries at the First Affiliated Hospital of Nanjing Medical University. Samples were divided into 4 groups with the same sex (all female): sun-protected + young(20-30 years old) group, sun-exposed + young(20-30 years old) group, sun-protected + Old(60-70 years old) group, sun- exposed + Old(60-70 years old) group. The sun-protected group consisted of breast or back skin areas and the sun-exposed group consisted of face or neck skin areas. The skin specimens were used for the Immunohistochemistry (IHC)-staining, western blot and PCR analyses. The present study was approved by the Local Ethics Committees of the First Affiliated Hospital of Nanjing Medical University (Nanjing, China) (Permit no. 2016-SRFA-033). The experiment information was informed to and agreed by all participants in this study.

### ELISA

The levels of type I collagen and type III collagen in serum were measured by using commercial ELISA kits (elabscience, Wuhan, China) following the instructions of manufacturers. The absorbance values were measured using a microplate spectrophotometer (Tecan, Switzerland) at 460 nm. The contents of collagen were determined according to the standard curves.

### qPCR

The skin tissues and HSF cells were homogenized and RNA was extracted with Trizol reagent (Invitrogen). After the RNA concentration determination and purification, 1 μg total RNA was applied for A-MLV reverse transcription kit (#K1622, thermo, Waltham, USA). Cycle threshold was measured by the iQSYBR Green PCR Master Mix. The relative abundance of Hrd1, IGF-1R, type I collagen and type III collagen expressions to the GAPDH expression were calculated by the 2^−ΔΔCt^ value using lightcycle®480 software. The sequence was listed at Table 1.

**Table 1:** The sequence of PCR primer.

**sequence name**	**resource**	**forward primer**	**reverse primer**
hrd1	human	CACTCTTCTTCTCTTCCTCAAATGT	GGCATACTCAAAGCCAAACAC
hrd1	mouse	TGCTCCTCTTCCTCAAATGTT	CTGTGATAAGCGTGGCTGAC
IGF-1R	human	AGGATATTGGGCTTTACAACCTG	GAGGTAACAGAGGTCAGCATTTT
IGF-1R	mouse	CCCAACCTCACAGTCATCCGTGGCT	CATTGTTGATGGTGGTCTTC
Col1	human	GAGGAGACTGGCAACCTGAA	AGGCGTGATGGCTTATTTGT
Col1	mouse	GCACGCCCAGTTTGGTAT	TCACACAAGTCCCTATCCATTA
Col3	human	CACGCAAGGCTGTGAGACTA	GACAAGATTAGAACAAGAGGAACAC
Col3	mouse	CCTGGCTCAAATGGCTCAC	TCCCTTCGCACCGTTCTT

### Immunohistochemistry

The HSF cells were assigned to control group, Ad-NC group, Ad-Hrd1 group, Ad-Hrd1 + UV group, UV group, Ad-NC + UV group, Ad-ShRNA-Hrd1 group, Ad-ShRNA-Hrd1 + UV group. 4 ×10^4^ cells were seeded on the six-well plate. 24 h later, HSF were incubated for 24 h with 2 × 10^5^ PFU Ad-NC, Ad-Hrd1, Ad-ShRNA-Hrd1 with serum-starved culture medium. The HSF cells were exposed to UVA 4J/cm^2^ and UVB 20mJ/cm^2^ irradiation. The irradiation distance between the UV source and HSF cell was 15 cm and the radiometer was also employed to control the irradiation concentration. After another incubation for 24 h with normal medium, the cells were stained with 4% paraformaldehyde for IHC.

The expression of Hrd1, IGF-1R, type I collagen and type III collagen in skin tissues and HSF cells were examined by immunohistochemistry. The paraffin samples were deparaffinized in xylene, rehydrated by graded ethanol, and heated in sodium citrate buffer. Then the slides were treated with 3% fresh H_2_O_2_. After blocking with 3% bovine serum albumin (BSA), the skin samples were incubated with the corresponding primary antibodies at 4 °C overnight. Following incubating with secondary antibody, the sections were further incubated with third antibody at 37 °C. Subsequently, the slides were exposed to DAB and then haematoxylin in the dark room. Finally, the section was dehydrated, mounted with neutral gum, and visualized under a microscope (Olympus, BX53, Japan).

### Western blot assay

The protein levels of Hrd1, IGF-1R, type I collagen and type III collagen in skin tissues and HSF were analyzed using Western blot analyses. The samples were lysed with RIPA buffer and centrifuged at 12000 g for 10 min and at 4°C. After the determination of protein concentration, equal amount of protein was subjected to SDS-PAGE and transferred onto the PVDF membrane. The membrane was blocked with 5% nonfat milk and followed by incubation with the appropriate dilutions of primary antibodies overnight at 4°C. Thereafter, the membranes were washed and incubated with HRP-conjugated secondary antibody at room temperature. The immunoreactive bands were observed using a gel imaging system and enhanced ECL system.

### Co-IP

After transfection with Ad-NC, Ad-shRNA-Hrd1, or Ad-Hrd1, HSF cells were exposed to MG-132 for 4 h. After determination of protein concentration, the samples were lysed with RIPA buffer containing protease/phosphatase inhibitors and then incubated with primary HRD-1 or IGF-1R antibodies. The target protein was then pulled down with protein-A/G magnetic beads. The samples were eluted and then incubated with IGF-1R or Hrd1 antibody. After the treatment with appropriated HRP secondary antibody, the immunoblots were then visualized with ECL system and gel imaging system.

### Statistical analysis

The results were presented as mean ± SD. Statistical analyses were conducted by one-way ANOVA followed by Tukey’s multiple comparison test using GraphPad 5.0 test. The p-value of less than 0.05 was regarded statistically significant.
